# A mito-centric view on muscle aging and function

**DOI:** 10.3389/fpubh.2023.1330131

**Published:** 2024-01-10

**Authors:** Johannes Burtscher, Barbara Strasser, Martin Burtscher

**Affiliations:** ^1^Institute of Sport Sciences, University of Lausanne, Lausanne, Switzerland; ^2^Ludwig Boltzmann Institute for Rehabilitation Research, Vienna, Austria; ^3^Faculty of Medicine, Sigmund Freud Private University, Vienna, Austria; ^4^Department of Sport Science, University of Innsbruck, Innsbruck, Austria

**Keywords:** aging, mitochondria, sarcopenia, exercise, diet, hypoxia, physical function

## 1 Introduction

Healthy lifestyles, such as those that include regular physical activity and a balanced diet, are a powerful means to prevent chronic disease and age-related functional decline. A common denominator of health improvements resulting from good exercise and diet habits is the optimization of metabolic processes. These processes include energy metabolism and, thus, the activity of mitochondria. Mitochondria represent hubs not only of cellular metabolism but also of the regulation of redox states, inflammatory response, and immunity, as well as many other cellular features (1). Mitochondria have emerged as highly flexible organelles that, quickly—and sometimes persistently—adapt to changing conditions in response to systemic or cellular challenges. Next to exercise and diets that promote mitochondrial health, transient exposures to environmental stressors, such as to altitude/hypoxia or extreme temperatures, also induce mitochondrial adaptations.

In this paper, we discuss how different systemic and cellular challenges trigger specific and overlapping mitochondrial responses that—under the right conditions—may translate into protective mitochondrial adaptations (2). We specifically focus on adaptations in skeletal muscle and sarcopenia, the age-related loss of skeletal muscle mass, strength, and function (3). Such responses rely on mechanisms such as mitochondrial stress responses and quality control; therefore, these mechanisms are believed to be required to maintain mitochondrial health (4). The resulting adaptations increase the capacity of mitochondria to respond to future stressors (e.g., altered oxygen or substrate availability), which otherwise might trigger pathological processes. Considering potential synergistic/anti-synergistic and complementary/competitive effects among lifestyle factors and environmental challenges on mitochondria, we argue that recommendations can be developed to increase performance, prevent sarcopenia, and improve healthy aging.

## 2 Mitochondrial medicine for muscle health in aging

### 2.1 Exercise interventions

Exercise represents a potent measure to foster healthy aging and to prevent and/or treat a large number of chronic diseases, including cardiovascular, pulmonary, neurological, metabolic, musculoskeletal diseases, and cancer (5). Those benefits, and in particular those promoted by endurance type training, are closely related to improved mitochondrial quality control (MQC, including mitophagy, the clearance of dysfunctional mitochondria), mitochondrial content, respiration, and dynamics in striated muscles (i.e., skeletal and heart muscle) (6–8). Regular exercise is thought to benefit mitochondria depending on the exercise type and intensity, although the specific determinants for mitochondrial improvements are still under debate, also due to the high diversity of exercise interventions and study populations (8, 9).

A recent systematic review reinforced the favorable effects of exercise training in older adults on mitochondrial quality, density, dynamics, oxidative, and antioxidant capacity, which varied according to the exercise type (9): While improvements of the mitochondrial antioxidant capacity appear to be important consequences of endurance exercise, resistance training seems to be particularly beneficial for mitochondrial density and dynamics.

Life-long high-volume exercise training specifically improved mitochondrial volume and network connectivity in skeletal muscle and associated oxidative capacity in older adults (10). Moreover, it preserved mitochondrial morphology, Ca^2+^ handling, and ATP production, contributing to the maintenance of skeletal muscle function in older individuals (11).

In subjects suffering from sarcopenia, more intense aerobic exercise protocols may more efficiently improve mitochondrial biogenesis (12); for example, exercise increased the mRNA levels of the mitochondrial biogenesis-related transcription factor peroxisome proliferator-activated receptor γ coactivator 1 α (*PGC-1*α*)* 10.2-fold at 80% of VO_2_max (maximum rate of oxygen consumption), but only 3.8-fold at 40% of VO_2_max (13). Low-volume high-intensity interval training (HIIT) represents a time-efficient alternative to improving skeletal muscle mass and cardiorespiratory fitness (CRF) in individuals, and even in octogenarians with co-morbidities, probably by increasing the mitochondrial oxidative phosphorylation capacity in skeletal muscle (14). Comparisons between HIIT, resistance training (RT), and the combination of HIIT and RT revealed that 12 weeks of HIIT enhanced mitochondrial content and resulted in protein changes in skeletal muscle indicative of increased mitochondrial fusion, while smaller effects were seen after combined training and (surprisingly) no effects after RT (15). These changes were associated with improved mitochondrial respiration, CRF, and insulin sensitivity in populations of untrained but lean young (18–30 years) and older (65–80 years) adults (15). Conversely, long-term RT (over 6 months) was found to considerably increase mitochondrial volume density in older individuals (16). In one recent study, 12 weeks of HIIT combined with L-citrulline supplementation increased markers of mitochondrial biogenesis, mitochondrial fusion and mitophagy in obese older adults and acted synergistically for improving muscle strength and muscle quality when compared with HIIT alone (17).

Taken together, these study findings indicate that exercise has the potential to improve or maintain mitochondrial content and health in skeletal muscle. This has been associated with healthy aging in older subjects provided that the training stimulus is appropriate, and higher intensities seeming to be more effective. Thus, it is crucial to individually tailor exercise interventions, considering individual conditions like existing diseases, exercise preferences and tolerability, training targets, as well as nutritional and supplementation strategies to support exercise-induced adaptations.

### 2.2 Dietary and combined interventions

The role of nutritional supplementation on sarcopenia risk and related outcomes (i.e., muscle strength, muscle mass, and performance) has been extensively summarized in previous reviews (18–21) highlighting the anti-aging potential of practicing a Mediterranean-style diet and demonstrating some evidence for the benefits of protein supplementation, especially in sarcopenic/frail older adults, when combined with RT. Frailty is a multidimensional condition that is closely related to sarcopenia (22) and mainly characterized by decreased functional reserves and stress resistance, and increased vulnerability (23). The widely used Fried frailty phenotype assesses physical frailty through five criteria: unintentional weight loss; weakness or poor handgrip strength; self-reported exhaustion; slow walking speed; and low physical activity (24).

Recently, the ProMuscle in Practice study demonstrated that increasing the amount of protein ingested per meal (≥25 g) along with twice-weekly progressive RT over a 12-week intensive support intervention was effective for counteracting sarcopenia in community-dwelling older adults who were frail or pre-frail based on Fried frailty criteria or who experienced strength loss (25). The recommended daily protein intakes are 1.0–1.2 g/kg body weight (BW) for healthy older individuals and 1.2–1.5 g/kg/BW for geriatric patients, containing ~2.5 g of leucine, to stimulate muscle protein synthesis (26). In addition, exercise and higher protein intake are recommended during weight loss, to avoid muscle wasting (27).

Caloric restriction, a lifestyle strategy to mitigate obesity and metabolic disease, which typically involves the consumption of 20–40% lower calories, shows beneficial effects on mitochondrial mass and function (28). However, this approach could also bring about unwanted reductions in lean mass, especially when the protein needs are not achieved, and may contradict dietary practices for optimizing skeletal muscle health in older persons (29). Thus, interventions to enhance the loss of fat while preserving muscle mass during energy restriction are of great importance to prevent sarcopenia in overweight older adults. Data indicate that, even in the presence of energy restriction, performance of RT with elevated daily protein ingestion (1.3 g/kg/BW) increases muscle protein synthesis and potentially supports muscle mass preservation during weight loss in obese older adults. In addition, short-term RT (over 2 weeks) stimulated mitochondrial protein synthesis as compared with energy restriction alone (30).

The few clinical trials of nutritional interventions on mitochondrial health in older healthy people or those with or at risk of malnutrition suggest that nutritional supplementation with branched-chain amino acids (BCAA) alone (31) or combined with 800 IU vitamin D3 per day (32) and omega-3 poly-unsaturated fatty acids (dosages from 3.3 to 3.9 g/day over a 4–6-month time period) (33, 34) may be useful in the prevention of sarcopenia. These strategies boost mitochondrial bioenergetic and redox capacities, potentially explaining the amelioration of muscular performance in older adults in the absence of exercise, which reflects the real-life situation of most community-dwelling older adults (18). Beta-hydroxy-beta-methylbutyrate (3 g/day), a metabolite of leucine, has been shown to concomitantly preserve muscle mass and mitochondrial gene expression in healthy older adults during 10 days of bed rest (35). Moreover, this supplementation improved mitochondrial content and dynamics over an 8-week RT rehabilitation period as compared with the placebo control (35).

Some micronutrients, such as zinc and selenium, may also contribute to mitochondrial health and reduce oxidative stress in sarcopenia, but the evidence is still too weak to promote these nutrients as treatments for sarcopenia (36).

Finally, probiotics may actively modulate the risk and progression of sarcopenia: Preclinical research findings suggest that *Lactobacillus casei* Shirota supplementation for 12 weeks enhances muscle function potentially through the gut–muscle axis via mitochondrial signaling (37, 38). Promoting a healthy gut microbiota also improves the bioavailability of dietary polyphenols. These compounds have been shown to benefit skeletal muscle cells and tissues, thus potentially representing effective components of a treatment strategy for reducing or reversing sarcopenia (39). Indeed, two studies detected improvements in mitochondrial density and oxidative phosphorylation capacity, accompanied by enhanced skeletal muscle morphology and better mobility in aged persons after 12 weeks of admission of resveratrol (500 mg/day) combined with exercise training (40, 41).

Taken together, the current evidence suggests that dietary interventions can be effective in the prevention and treatment of sarcopenia by improving various aspects of mitochondrial health. Adherence to a Mediterranean diet, which favors a high intake of proteins, fibers, and polyphenols, and nutritional supplementation with BCAA, omega-3 polyunsaturated fatty acids, and vitamin D should be considered in older adults to support exercise-induced adaptations and muscle health. Overall, it can be concluded that the combination of diet and exercise interventions due to synergistic and complementary effects may be is the most effective approach to protect mitochondria to ameliorate sarcopenia.

### 2.3 Altitude and hypoxia

Epidemiological studies reveal that living at moderate altitudes (1,000–2,000 m) may increase human life expectancy (42, 43). Reduced mortality from cardiovascular diseases and certain cancer types are thought to be main mediators of this effect, and they thought to be a consequence of the lower oxygen partial pressure, and thus reduced oxygen availability (hypoxia), at these altitudes (43). This hypothesis is supported by evidence that exposure to mild chronic continuous environmental hypoxia extends the lifespan of various species, including worms (44), fruit flies (45), and mice (46).

Accumulating evidence suggests that brief and repeated exposures to mild or moderate hypoxia (hypoxia conditioning, HC) also induce physiological and cellular adaptations which protect individuals from subsequent, more severe hypoxic or ischemic insults and possibly from age-related diseases (47, 48). In contrast to the potential beneficial impact on healthy aging and life expectancy conferred by exposure to mild or moderate hypoxia, exposure to more severe hypoxia may even accelerate aging, potentially due to the augmentation of oxidative stress, inflammation, and mitochondrial dysfunction (48). Thus, major health benefits from hypoxia exposure may not result from hypoxia *per se* but rather from adaptations initiated by exposures to hypoxia at appropriate intensities, durations, and frequencies (49).

Mitochondria are key to adaptations involved in the induction of cellular stress responses, the upregulation of antioxidant pathways, and the optimization and reduction of oxidative metabolism rates (4, 50). Interventional studies have revealed the preventive and therapeutic effects and promotion of healthy aging by HC by, for example, improving exercise tolerance and cognitive performance in healthy older adults or those with pre-existing cardiovascular, pulmonary (51), or age-related neurological deficits (52). For example, after 3 weeks of intermittent hypoxia, VO_2_max increased by 6.2% in older men with and without coronary artery disease while no change was observed in the normoxic control (53).

Moreover, exercise training in athletes (twelve high-intensity treadmill sessions over 6 weeks, in addition to regular trainings) under normobaric hypoxic conditions (FiO_2_: 14.5%, 3,000 m) increased the gene expression of the mitochondrial biogenesis regulators *PGC-1*α and *transcription factor A* and elevated mitochondrial enzyme activity (i.e., of citrate synthase and cytochrome oxidases 1 and 4) (54). A recent meta-analysis revealed greater improvements in the body fat and body mass index of middle-aged and older adults when exercise was performed under normobaric hypoxic conditions as compared to normoxic conditions (55). The authors suggest that changes in cellular energy production and mitochondrial protein synthesis may be potential mechanisms associated with modifications in body composition (55). However, whether exercising in hypoxia benefits older people more than exercising in normoxia remains to be elucidated. A recent study in sedentary older individuals found no differences in mitochondrial and functional outcomes between these modalities after 8 weeks of aerobic exercise (56).

In summary, together with exercise and dietary interventions, HC represents a promising strategy to counteract skeletal and cardiac muscle dysfunction and conditions of sarcopenia, and thus to promote healthy aging. Which HC programs optimally improve specific mitochondrial functions and muscle health remain to be identified and will need to consider individual circumstances (e.g., physical and mental performance capabilities, co-morbidities, pharmacological therapy, and responsiveness to hypoxia exposure). A selection of studies linking exercise, dietary, hypoxia and combined interventions to mitochondrial and muscle or fitness outcomes are summarized in Table 1.

**Table 1:** Selected lifestyle interventions targeting mitochondria in muscle aging.

**Intervention and population**	**Mitochondrial outcomes**	**Muscle or fitness outcomes**	**References**
**Exercise interventions**			
Four-week HIIT, ≈15 min/week (100–115% of maximal workload) in octogenarians (81.2 ± 0.6 years) with comorbidities	Improved mitochondrial oxidative phosphorylation capacity: increased activities of the mitochondrial enzymes citrate synthase and complexes II and III of the respiratory system	Increased muscle protein synthesis, cardiorespiratory fitness and fat-free mass	(14)
Twelve-week HIIT, resistance, or combined training in young (18–30 years) and older (≥65) sedentary adults	Increased mitochondrial volume and number, higher protein levels of the mitochondrial fusion factor OPA1 and improved mitochondrial respiration by HIIT	Improved cardiorespiratory fitness and fat-free mass in all training groups	(15)
Eight-week resistance, endurance or combined training in young (18–30 years) and older (≥65) sedentary adults	Improved mitochondrial respiration in combined training groups (young and older)	Combined training resulted in the most robust improvements in muscle strength, quality, and fitness	(57)
**Dietary interventions**			
BCAA supplementation of older malnourished patients (>80 years) for 8 weeks	Improved mitochondrial biogenesis and fusion and lower levels of oxidative stress	Improvements in nutritional status, muscle mass, strength and performance	(31)
Supplementation of older adults (>65) with/at risk of undernutrition for 12 weeks: whey and casein protein, ursolic acid, free BCAA and vitamin D	Upregulated expression of gene clusters (microarray) related to oxidative phosphorylation, mitochondrial functioning, and mitochondrial biogenesis	Improved walking performance	(32)
Omega-3 poly-unsaturated fatty acid supplementation (1.86 g/day EPA, 1.5 g/day DHA) for 6 months in older (60–85 years) adults	Small, upregulated expression of gene clusters (microarray) related to mitochondrial functions	Improved muscle mass and function	(34)
**Combined exercise and dietary interventions**			
Citrulline supplementation (10 g/day) and HIIT for 12 weeks in obese older (67.2 ± 5.0 years) adults vs. placebo (same exercise program)	Increased mitochondrial biogenesis, mitochondrial fusion, and mitophagy in both groups	Improved lean mass, muscle power, and function in both groups; greater increase in muscle strength and quality	(17)
Two-week energy restriction (−300 kcal/day, 1.3 g/kg/day protein) vs. energy restriction plus resistance training in obese older (66 ± 4 years) men	Increased mitochondrial and myofibrillar protein synthesis by resistance training	Preserved muscle mass during weight loss by resistance training	(30)
Beta-hydroxy-beta-methylbutyrate (3 g/day) supplementation during 8-week resistance training in 60–76 years old subjects after 10 days bed rest	Increased protein levels of oxidative phosphorylation components and of mitochondrial fusion (mitofusin 2) and fission (DRP1) factors	Preserved muscle mass	(35)
Resveratrol (500 mg/day) and combined resistance/endurance training for 12 weeks in 65–80 years old subjects vs. placebo (same exercise program)	Increased mitochondrial density, higher transcription levels of mitochondrial pro-fusion factors	Increased muscle fatigue resistance, mean fiber area and muscle torque/power	(40)
**Exercise in hypoxia**			
Sedentary adults (62 ± 6 years) trained 3 × per week for 8 weeks in normobaric hypoxia (15%) vs. normoxia on a bicycle ergometer	No differences in markers of mitochondrial content and oxidative capacity (activities of citrate synthase and components of the mitochondrial respiratory system)	Similar improvements in muscle metabolism (lactate, fat and carbohydrate oxidation) and in power output after hypoxic and normoxic training	(56)

BCAA, branched-chain amino acids; DHA, docosahexaenoic acid; EPA, eicosapentaenoic acid; HIIT, high-intensity interval training; OPA1, optic atrophy 1.

## 3 Discussion

Increasing mitochondrial deficits (58), the associated oxidative stress (58), and inflammatory processes (59) are central processes in aging. Accordingly, mitochondrial health and the associated oxidative capacity also declines with age in skeletal muscle. This decline is correlated to reduced muscle performance and CRF (60, 61). It is thus plausible that aging mitochondria are involved in the development of age-related sarcopenia, although the specific concerned mitochondrial deficits (e.g., oxidative phosphorylation, biogenesis, dynamics, quality control) remain to be elucidated (62). Importantly, direct data on mitochondrial outcomes of lifestyle-interventions in sarcopenia are scarce and experimental confirmation on potential preventive and symptomatic benefits are urgently required.

The overlapping but also seemingly differential mitochondrial benefits of different lifestyle interventions might be harnessed to design optimized mixed lifestyle interventions that counteract the development of sarcopenia or alleviate its symptoms. These interactions among lifestyle factors and their results on mitochondrial activity, however, are still poorly known and controversially discussed. Different exercise modalities and intensities, for example, may differentially improve the mitochondrial biogenesis (resistance training, moderate endurance training) and dynamics (resistance training), antioxidant capacity (moderate endurance training), quality control/mitophagy (moderate endurance training), or oxidative phosphorylation capacity (intensive endurance training) (8, 9).

Like exercise, a mild caloric restriction and certain nutrients may help to preserve specific facets of mitochondrial health and might be suitable as a counteracting strategy for sarcopenia. These include the use of beta-hydroxy-beta-methylbutyrate supplements, which seem to effectively promote mitochondrial density and dynamics (35). In combination with exercise, resveratrol also appears to improve mitochondrial density and oxidative phosphorylation (40, 41).

**Figure 1:**
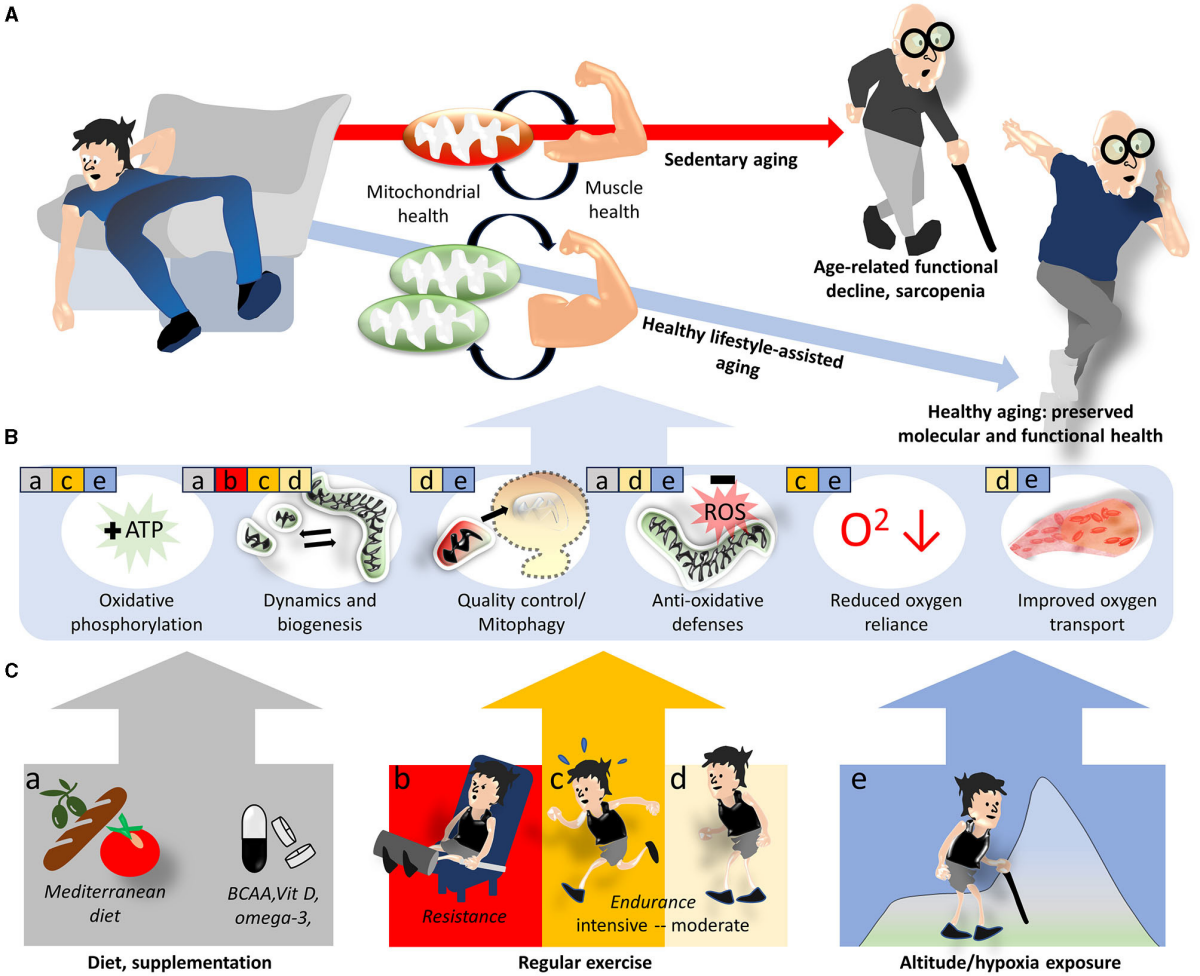
Lifestyle interventions modulating mitochondrial and skeletal muscle health to prevent sarcopenia. Sedentary aging is associated with increasing mitochondrial deficits and functional decline of skeletal muscle and favors the development of sarcopenia, while the adoption of a healthy lifestyle provides some protection **(A)**. On the molecular level, this protection is believed to be mediated by improvements in various mitochondrial functions and related oxygen utilization factors **(B)**. Specific aspects of mitochondria and oxygen utilization discussed in the text are indicated by colored squares and letters a–e in **(B)**, where the colors/letters correspond to lifestyle/environmental factors shown in **(C)**.

The controlled variation in hypoxia levels (by climbing to different altitudes, spending time in hypoxia chambers/tents, breathing of defined gas mixtures, or performing breathing exercises) may also modulate specific mitochondrial functions, depending on the severity, duration, and frequency of the exposure (50). Cellular adaptations to hypoxia include increased oxidative phosphorylation efficiency and antioxidative capacities, but also enhanced cellular oxygen supply due to the improved oxygen transport in the blood (e.g., as a result of erythropoietin upregulation) and angiogenesis, as well as glucose transport and glycolysis upregulation, which reduce the reliance of ATP production on oxygen levels (50).To take full advantage of the potentially complementary and synergistic benefits of exercise, dietary strategies and hypoxia exposure, the distinct effects of these interventions need to be better understood (Figure 1).

Very few studies report small or no effects of higher physical activity levels and/or healthy dietary behaviors, such as Mediterranean diet, on sarcopenia prevalence [e.g., (63)], suggesting limitations of the preventive potential of healthy lifestyles.

Specifically, sarcopenic or malnourished older adults tend to develop an anabolic resistance to three fundamental anabolic stimuli [i.e., insulin signaling, BCAA (primarily leucine) blood concentration, and physical activity]. For these older people (64), individually optimized dietary protein intake combined with RT are required to maintain or improve muscular strength and mitochondrial function with aging. Physical activity, and predominantly endurance exercise, often potently counteracts sedentary aging associated with mitochondrial dysfunction, insulin resistance and obesity. But combinations with adequate dietary strategies, RT and hypoxia can further optimize mitochondrial health and muscle performance. Well-calibrated RT benefits almost all older people (65) and reduces the risk of sarcopenia in older adults adhering to aerobic moderate-to-vigorous physical activity guidelines even further (66).

Based on the high complexity of outcomes in lifestyle changes, the investigation of combined approaches (e.g., diet and exercise interventions) are challenging and individualized combinations of different training types and dietary regimes accompanied by monitoring and continuous program adaption will be important to guarantee success. Person-centered strategies are especially important for vulnerable populations to ensure exercise and/or hypoxia benefits and an appropriate nutritional status, while balancing these factors with associated risks (injury risk, oxidative stress, immune system consequences, and inflammation).

Other lifestyle and environmental factors that have not been considered in this review but may be similarly important (e.g., sleep or heat/cold acclimatization) also require further study. The complex physiological consequences of lifestyle and environmental changes also complicate efforts to compare the associated mitochondrial effects. The specific strategy outcomes, meanwhile, are determined by the application modalities (or supplement type), dose, and individual characteristics of the recipient (e.g., genetic makeup, fitness and health status). However, availability of experimental (e.g., OMICS) approaches together with increasingly powerful analytical/bioinformatic tools will pave the way for the development of a person-centered lifestyle medicine that can prevent sarcopenia and other age-related diseases.

## Author contributions

JB: Writing—original draft, Writing—review & editing. BS: Writing—original draft, Writing—review & editing. MB: Writing—original draft, Writing—review & editing.
